# Ameobal Pathogen Mimivirus Infects Macrophages through Phagocytosis

**DOI:** 10.1371/journal.ppat.1000087

**Published:** 2008-06-13

**Authors:** Eric Ghigo, Jürgen Kartenbeck, Pham Lien, Lucas Pelkmans, Christian Capo, Jean-Louis Mege, Didier Raoult

**Affiliations:** 1 URMITE CNRS UMR 6236 - IRD 3R198, Université de la Méditerranée, Marseille, France; 2 Abteilung für Zellbiologie, Deutsches Krebsforschungszentrum Heidelberg, Heidelberg, Germany; 3 Institute of Applied Physics, ETH Zürich, Zürich, Switzerland; 4 Institute for Molecular Systems Biology, ETH Zürich, Zürich, Switzerland; Harvard Medical School, United States of America

## Abstract

Mimivirus, or *Acanthamoeba polyphaga mimivirus* (APMV), a giant double-stranded DNA virus that grows in amoeba, was identified for the first time in 2003. Entry by phagocytosis within amoeba has been suggested but not demonstrated. We demonstrate here that APMV was internalized by macrophages but not by non-phagocytic cells, leading to productive APMV replication. Clathrin- and caveolin-mediated endocytosis pathways, as well as degradative endosome-mediated endocytosis, were not used by APMV to invade macrophages. Ultrastructural analysis showed that protrusions were formed around the entering virus, suggesting that macropinocytosis or phagocytosis was involved in APMV entry. Reorganization of the actin cytoskeleton and activation of phosphatidylinositol 3-kinases were required for APMV entry. Blocking macropinocytosis and the lack of APMV colocalization with rabankyrin-5 showed that macropinocytosis was not involved in viral entry. Overexpression of a dominant-negative form of dynamin-II, a regulator of phagocytosis, inhibited APMV entry. Altogether, our data demonstrated that APMV enters macrophages through phagocytosis, a new pathway for virus entry in cells. This reinforces the paradigm that intra-amoebal pathogens have the potential to infect macrophages.

## Author Summary

The giant (750 nm) double-stranded DNA virus *Acanthamoeba polyphaga mimivirus* (APMV) is likely responsible for pneumonia. We demonstrate here that APMV was internalized by macrophages but not by non-phagocytic cells, leading to productive replication. We also show that APMV invaded macrophages through phagocytosis. This is the first evidence that a virus is internalized by macrophages via a mechanism normally used by bacteria and parasites. This finding adds a supplementary pathway to already known strategies used by viruses to enter cells. This underlines that intra-amoebal pathogens also infect macrophages. Finally, we can hypothesize that APMV replicates within alveolar macrophages, leading to human and murine pneumonia.

## Introduction

*Acanthamoeba polyphaga mimivirus* (APMV), isolated from amoebae cultures, was identified for the first time in 2003 in our laboratory [1]. This double-stranded DNA virus is classified in the *Mimiviridae* family [1]. AMPV is probably responsible for pneumonia as suggested in human pneumonia [2], and evidenced in APMV-inoculated mice [3]. APMV is a giant icosahedral enveloped virus surrounded by fibrils about 750 nm in diameter [4], a morphology that is reminiscent of that of Iridoviruses, Asfarviruses and Phycodnaviruses. It is larger than that of small mycoplasma, such as *Ureaplasma urealyticum*, and is comparable in size to that of intracellular bacteria, such as *Rickettsia conorii* or *Tropheryma whipplei* [1],[2]. It has been suggested that APMV infects ameoba by phagocytosis, but this has not been demonstrated [5].

**Figure 1:**
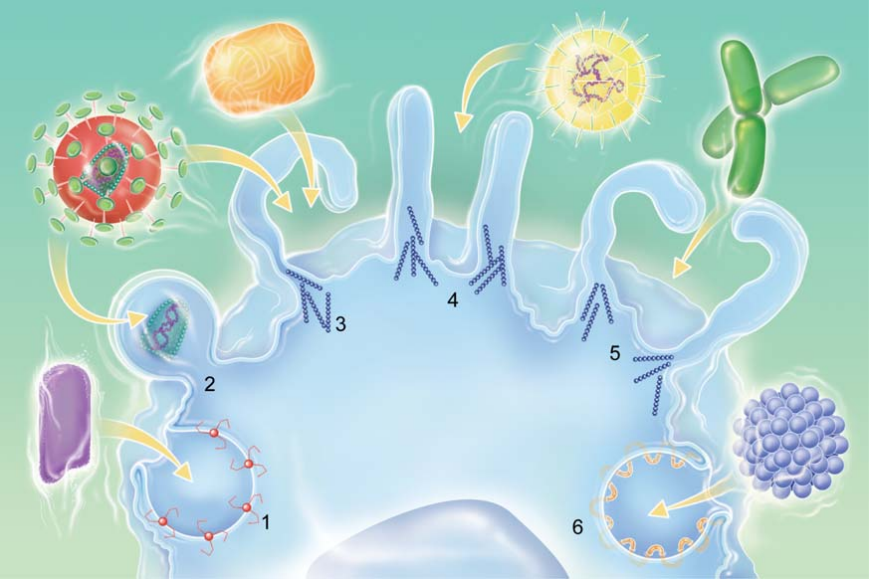
Multiple portals of virus entry into mammalian cells. (1) Clathrin-mediated entry (i.e. Vesicular Stomatitis virus), (2) fusion-entry (i.e. Human immunodeficiency virus), (3) macropinocytosis-mediated entry (i.e Vaccinia virus), (4) phagocytosis-like-mediated entry (i.e. Herpes simplex virus), (5) phagocytosis-mediated entry (i.e. bacteria), (6) caveolin-mediated entry (i.e. Simian virus 40).

Viruses have evolved a variety of mechanisms to deliver their genes and accessory proteins into host cells. The first step of viral invasion consists of passing through the host cell's plasma membrane, which is a major barrier for invading agents. Several internalization pathways have been described, differing in the size of the endocytic vesicles, the nature of the cargo and the mechanism of vesicle formation. They include clathrin-mediated endocytosis, caveolin-mediated endocytosis, macropinocytosis and phagocytosis [6] (**Figure 1**). Clathrin-mediated endocytosis is characterized by the clustering of ligated transmembrane receptors into clathrin-coated pits of about 120 nm. Vesicular stomatitis virus and Semliki Forest virus enter host cells through clathrin-mediated endocytosis [7],[8], although vesicular stomatitis virus might also entry host cells through a degradative endosome-mediated endocytosis [9]. Endocytosis can also involve caveolae pits, which are small vesicles of 50 to 80 nm enriched with caveolin, cholesterol and sphingolipids; simian virus uses this route to enter host cells [10] (**Figure 1**). The criterion of particle size is not sufficient to predict the mechanism of internalization, since clathrin and caveolin are also involved in the internalization of particles larger than 1 µm by macrophages [11],[12]. Macropinocytosis traps large amounts of macromolecules and fluid. This endocytic pathway, which is independent of receptors and dynamin-II, is associated with actin-dependent plasma membrane ruffling [7],[13] and is inhibited by amiloride analogs [14],[15]. Macropinocytosis leads to the formation of macropinosomes, which are large vesicles (>1 µm) characterized by the presence of rabankyrin-5 [16]. It has been shown that, in certain conditions, vaccinia virus and human immunodeficiency virus are able to use macropinocytosis to invade host cells [17],[18]. Phagocytosis, which is restricted to professional phagocytes, consists of the uptake of large particles (>500 nm), microorganisms, cell debris and apoptotic cells. It is initiated by the interaction of cell surface receptors, such as mannose receptors, Fc receptors and lectin receptors, with their ligands, which are present at the particle surface, and leads to particle internalization through an actin-dependent mechanism [19]. In contrast to macropinocytosis, phagocytosis requires dynamin-II, a ubiquitously expressed GTPase that has a critical role in the scission of forming clathrin-coated endocytic vesicles from the plasma membrane and the formation of phagosomes. Indeed, dominant negative forms of dynamin-II inhibit phagocytosis at the stage of membrane extension around particles [6],[7],[20]. Herpes simplex virus infects target cells, primary human corneal fibroblasts and Chinese hamster ovary cells, through a phagocytosis-like mechanism [21], but such a mechanism is not restricted to professional phagocytes.

In the present study, we provide evidence that APMV particles were internalized by macrophages, but not by non-professional phagocytic cells, leading to a productive cycle of virus replication. We also demonstrate that APMV invaded macrophages through phagocytosis. This is the first evidence that a virus is internalized by macrophages via a mechanism normally used by bacteria and parasites.

## Results

### Virus entry into macrophages

**Figure 2:**
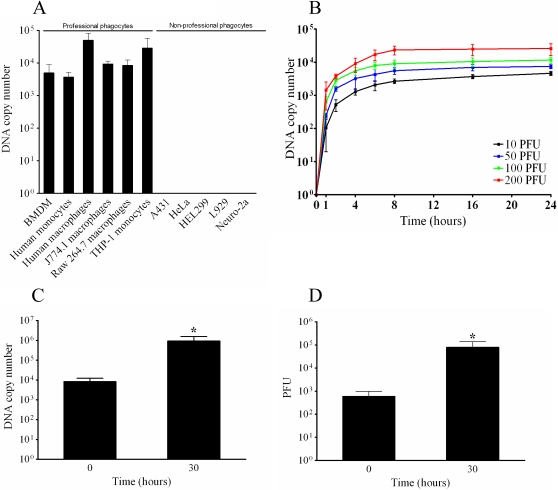
Cell infection with APMV. (A) Cells were incubated with APMV (50 PFU/cell) for 6 hours, extensively washed, and the number of viral DNA copies was determined by qPCR. (B) Macrophages were infected with different concentrations of APMV for different periods of time. After washing, the number of viral DNA copies was determined by qPCR. (C) Macrophages were infected with APMV (50 PFU/cell) for 6 hours (t = 0). After washing, macrophages were incubated for an additional period of 30 hours and viral particles were recovered. The number of viral DNA copies was determined by qPCR. (D) The infectivity of APMV particles was evaluated by incubating viral particles with amoebae and PFU were scored. The results are the mean±SD of 5 experiments. *p<0.05.

In first intention, we observed that APMV is internalized by professional phagocytes, but not by non-professional phagocytic cells (**Figure 2A**). Indeed, APMV enters human myeloid cells, including circulating monocytes, monocyte-derived macrophages and THP-1 myelomonocytic cells. APMV also enters mouse myeloid cells, such as bone marrow-derived macrophages (BMDM), RAW 264.7 macrophages and J774A.1 macrophages. In contrast, APMV was not internalized by non-phagocytic cells, including human lung fibroblast HEL299, mouse fibroblast L929 cells, human epithelial A431 or HeLa cells, or Neuro-2a mouse neuronal cells. The absence of APMV internalization by non-phagocytic cells was not due to delayed uptake or a low viral burden, since APMV particles were not internalized when the incubation time and the viral load were increased. Our results clearly show that APMV infected phagocytic cells but was unable to infect non-professional phagocytes. For technical reasons, we decided to use RAW 264.7 macrophages to investigate the mechanism of APMV entry.

The uptake of APMV by RAW 264.7 macrophages was assessed by real time PCR. With a PFU-to-cell ratio of 10∶1, APMV uptake was detected after 2 hours and increased thereafter. It reached a plateau after 8 hours that was maintained for 24 hours (**Figure 2B**). The uptake increased in a dose-dependent manner. Using a PFU-to-cell ratio of 50∶1, viral DNA copies were detected after 1 hour of incubation with macrophages and increased to a plateau after 8 hours. When macrophages were infected with APMV using PFU-to-cell ratios of 100∶1 and 200∶1, the time course of infection was similar to those observed with lower doses of viral particles. The number of viral DNA copies associated with macrophages was roughly proportional to the infective doses of APMV (**Figure 2B**). In subsequent experiments, a dose of 50 PFU per cell was used to infect cells for 6 hours: this time was defined as t = 0 for further experiments.

The infection cycle of APMV within RAW 264.7 macrophages was also evaluated. Macrophages were infected for 6 hours (t = 0), extensively washed to discard unbound viruses, and incubated for an additional period of 30 hours. As determined by real time PCR, the number of viral DNA copies increased significantly (p<0.05) during this period (**Figure 2C**). APMV was cytopathogenic for macrophages, since only 23±13% of macrophages were viable 30 h after infection. The increased number of viral DNA copies was associated with cycles of productive APMV replication. Indeed, serial dilutions of macrophage extracts were added to amoebae and amoebae lysis was determined. APMV particles produced by macrophages replicated within amoebae (**Figure 2D**), indicating that APMV internalization by RAW 264.7 macrophages led to a productive cycle of viral infection. Taken together, these data suggest that APMV infected the macrophages and led to a productive cycle of viral infection.

### Caveolae and clathrin are not involved in APMV uptake

**Figure 3:**
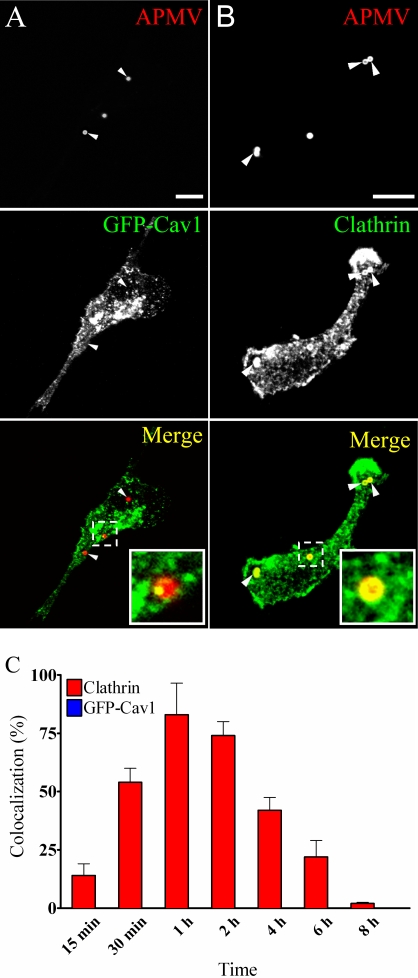
APMV particles colocalize with clathrin, but not with caveolin-1. (A) RAW 264.7 macrophages overexpressing GFP-caveolin-1 (GFP-Cav1) were incubated with APMV particles (50 PFU/cell) for 15 min. Viral particles were visualized by indirect immunofluorescence and laser scanning microscopy. The lack of colocalization with GFP-caveolin-1 was demonstrated by merging fluorescent images. (B) RAW 264.7 macrophages were incubated with APMV particles (50 PFU/cell) for 1 hour. The colocalization of viral particles with Alexa 488-conjugated clathrin antibodies was determined. Merged images showed the colocalization of APMV particles with clathrin. Inset confirms the colocalization of clathrin with viral particles. (C) RAW 264.7 macrophages were incubated with APMV particles (50 PFU/cell) for different periods. The number of APMV particles that colocalized with clathrin was scored. The results are expressed as the percentage of AMPV particles that colocalized with clathrin and are the mean±SD of 4 experiments. Scale bars represent 3 µm.

**Figure 4:**
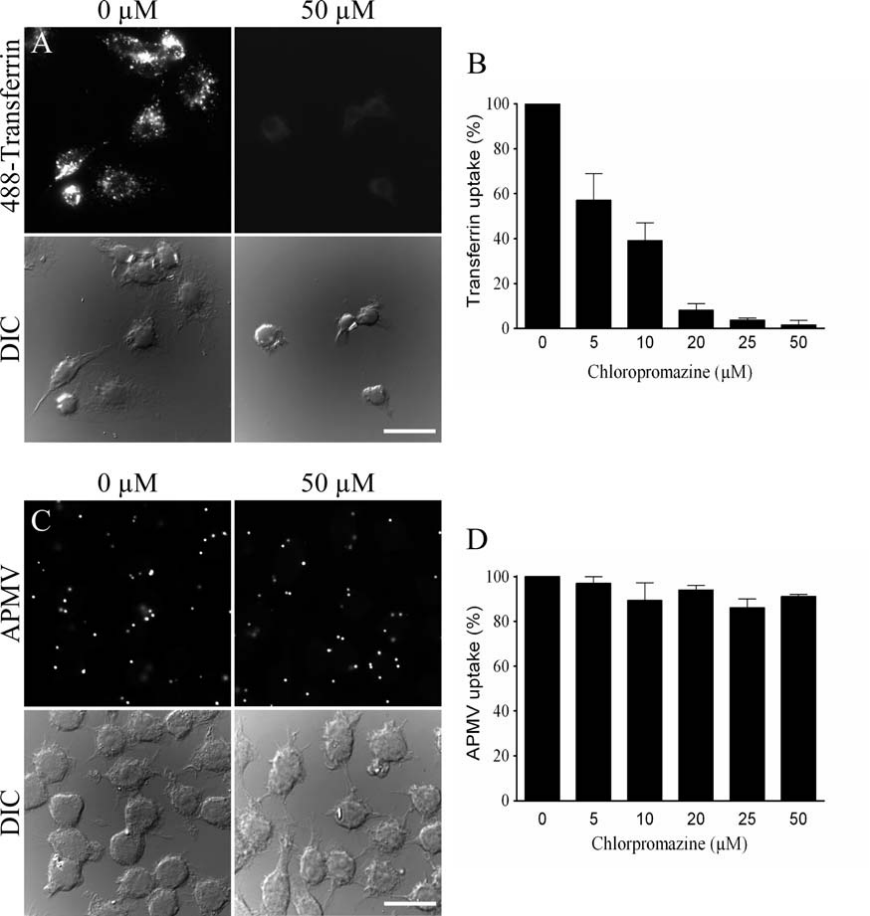
Role of clathrin in APMV internalization. (A) RAW 264.7 macrophages were pretreated with 50 µM chlorpromazine for 30 min and incubated with 50 µg/ml Alexa 488-conjugated transferrin for 15 min. The intracellular distribution of fluorescent transferrin was studied in control macrophages (left panels) and in chlorpromazine-pretreated macrophages (right panels). (B) Macrophages were pretreated with different concentrations of chlorpromazine and incubated with fluorescent transferrin. The results, expressed as the percentage of transferrin uptake relative to the control, are the mean±SD of 3 experiments. (C) Macrophages were pretreated with 50 µM chlorpromazine for 30 min before a 6-hour infection with APMV (50 PFU/cell) in the presence of 20 µM monensin to limit chlorpromazine toxicity. APMV particles were revealed by indirect immunofluorescence. Their intracellular distribution was studied in control macrophages (left panels) and in chlorpromazine-pretreated macrophages (right panels). (D) Macrophages were treated with different concentrations of chlorpromazine and incubated with APMV particles. Viral particles were visualized by immunofluorescence. The results, expressed as the percentage of APMV uptake relative to the control, are the mean±SD of 5 experiments. Scale bars represent 50 µm.

**Figure 5:**
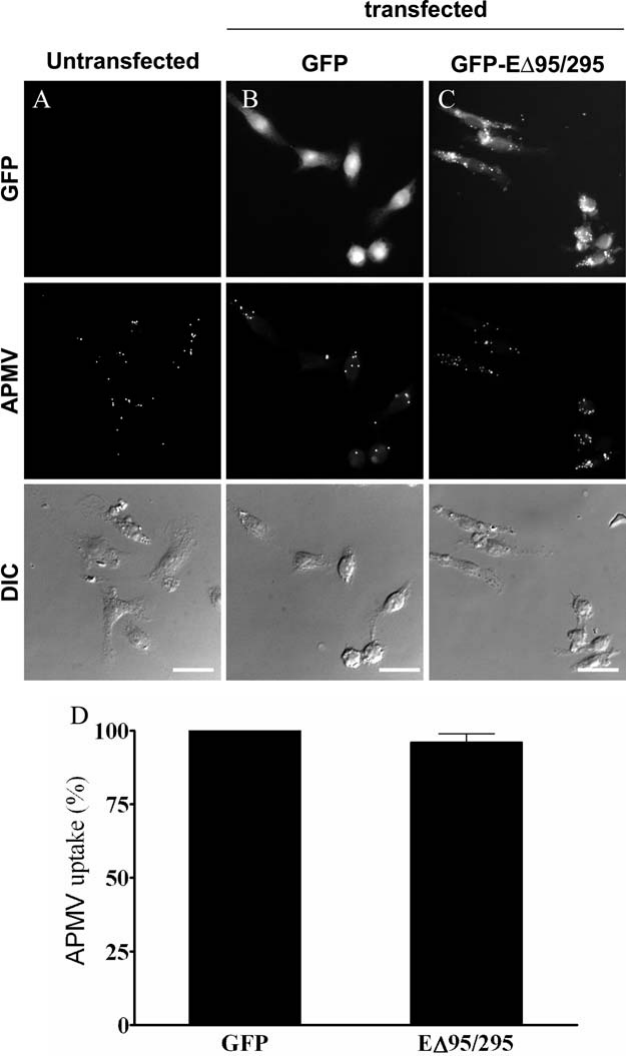
Dominant negative mutant of Eps15 does not inhibit APMV internalization. RAW 264.7 macrophages (A), macrophages transiently transfected with GFP (B) and dominant-negative mutant of Eps15 (EΔ95/295) (C) were infected with APMV (50 PFU/cell) for 6 hours. Viral particles were visualized by immunofluorescence (middle panels). Viral particles were associated with macrophages transfected with EΔ95/295. (D) The number of APMV particles internalized was scored. The results, expressed as the percentage of APMV uptake relative to the control, are the mean±SD of 4 experiments. Scale bars represent 25 µm.

As clathrin- and caveolae-mediated endocytic pathways are usually used by viruses to enter and infect cells, their role in APMV entry was investigated by immunofluorescence and confocal microscopy. Macrophages overexpressing GFP-caveolin-1 (GFP-Cav1) were incubated with APMV for 15 min; APMV particles did not colocalize with GFP-caveolin-1 (**Figure 3A**). The lack of colocalization of viral particles with GFP-caveolin-1 was not due to delayed acquisition of GFP-caveolin-1, since any colocalization was observed over a period of 8 hours. However, APMV particles colocalized with clathrin (**Figure 3B**). After 15 min of incubation, 14±5% of viral particles colocalized with clathrin. This percentage increased after 30 min, reached a maximum at 1 hour (83±14%) and progressively decreased to reach 2±0.5% at 8 hours (**Figure 3C**). To assess the role of clathrin in APMV internalization by macrophages, we used chlorpromazine, an inhibitor of clathrin-mediated endocytosis [22]. The activity of chlorpromazine was tested through the inhibition of transferrin uptake. Chlorpromazine inhibited transferrin uptake in a dose-dependant manner with 50 µM chlorpromazine mediating maximum inhibition (**Figure 4A** **and** **Figure 4B**). Chlorpromazine-treated macrophages were incubated with APMV for 6 hours in the presence of 20 µM monensin to limit cell toxicity resulting from long-term incubation with chlorpromazine [22]. APMV uptake by RAW 264.7 macrophages was not affected by chlorpromazine regardless of its concentration (**Figure 4B** **and** **Figure 4C**). In these experimental conditions, chlorpromazine at concentrations between 5 and 50 µM did not affect the viability of macrophages (**Table 1**). As an alternative approach to the use of inhibitory drugs, we used the expression of a dominant negative mutant of Eps15 (EΔ95/295) that inhibits clathrin-dependent endocytosis [23]. RAW 264.7 macrophages were transfected with a construct encoding a GFP fusion protein of the Eps15 deletion mutant (GFP-EΔ95/295) and a construct encoding GFP as control. First, GFP-EΔ95/295 activity was tested through the inhibition of transferrin uptake (**Figure S1)** [24]. The expression of GFP did not affect the transferrin uptake ability of macrophages (**Figure S1A and Figure S1B**) since transferrin was completely internalized by GFP-expressing macrophages as compared to untransfected cells. In contrast, overexpression of GFP-EΔ95/295 inhibited transferrin uptake by RAW 264.7 macrophages (**Figure S1C and Figure S1D**) while GFP-overexpressing macrophages internalized transferrin (**Figure S1B and Figure S1D**). Second, RAW 264.7 macrophages overexpressing GFP-EΔ95/295 or GFP were infected with APMV for 6 hours, and APMV uptake was determined by immunofluorescence. The expression of GFP had no effect on APMV internalization since 94.5±4% of APMV particles were internalized by GFP-expressing macrophages as compared to untransfected cells (**Figure 5A** **and** **Figure 5B**). Macrophages overexpressing GFP-EΔ95/295 also internalized APMV (**Figure 5C**): 96±3% of APMV particles were internalized by macrophages compared to GFP-expressing macrophages (**Figure 5D**). Taken together, these data suggest that caveolin-1 and clathrin are not involved in APMV uptake despite the colocalization of viral particles with clathrin.

**Table 1:** Viability of drug-treated macrophages.

Drugs (μM)		Cell viability (%)
Chlorpromazine * ^(1)^	0	100
	5	95±2
	10	93±6
	20	95±3
	25	93±5
	50	86±6
Cytochalasin D ** ^(2)^	0	100
	0.2	94±3
	1	91±5
	5	96±4
Ly294002 ** ^(2)^	0	100
	10	91±6
	50	96±2
	100	88±8
EIPA ** ^(1)^	0	100
	10	95±4
	25	96±2
	50	90±3
	100	85±7
	200	75±6

*: Chlorpromazine-treated RAW 264.7 macrophages were incubated with APMV for 6 hours in the presence of 20 μM monensin to limit the long-term cell toxicity due to chlorpromazine.

**: RAW 264.7 macrophages were pretreated with various concentrations of cytochalasin D, LY294002 or EIPA for 30 min, and incubated with APMV for 6 hours in the presence of the inhibitor. Macrophage viability was evaluated using the EZ4U kit. The results, expressed as the percentage of viable cells, are the mean±SD of 3 ^(2)^ or 5 ^(1)^ experiments.

### Degradative endosomes are not involved in APMV uptake

**Figure 6:**
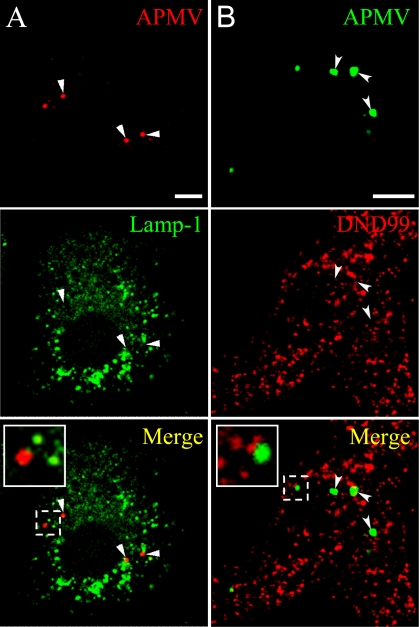
Lack of colocalization of APMV particles with Lamp-1 and lysotracker red DND99. (A) RAW 264.7 macrophages were incubated with APMV particles (50 PFU/cell) for 15 min. Viral particles and Lamp-1 were visualized by indirect immunofluorescence and laser scanning microscopy. The lack of colocalization of viral particles with Lamp-1 was demonstrated by merging fluorescent images. (B) RAW 264.7 macrophages loaded with lysotracker red DND99 were incubated with APMV particles (50 PFU/cell) for 15 min. The colocalization of viral particles with lysotracker red DND99 was determined. Merged images showed that APMV particles did not colocalize with lysotracker red DND99. Insets confirmed the lack of colocalization of viral particles with Lamp-1 or lysotracker red DND99. Scale bars represent 3 µm.

As the degradative endosome-mediated endocytic pathway might be used by viruses to enter and infect cells, its role in APMV entry was investigated using Lamp-1 (Lysosomal-associated membrane protein-1), a marker of late endosomes and lysosomes, and the lysotracker red DND99, a weakly basic amine that selectively accumulates in compartments with low pH such as lysosomes. RAW 264.7 macrophages were incubated with APMV for different periods of time, and the colocalization of the organisms with Lamp-1 and lysotracker red DND99 was assessed by immunofluorescence and confocal microscopy. APMV particles did not colocalize with Lamp-1 (**Figure 6A**) and lysotracker red DND99 (**Figure 6B**). This was not due to delayed colocalization of viral particles with Lamp-1 and lysotracker red DND99 since any colocalization was observed over a period of 6 hours (**Table 2**). These results suggest that APMV entry into macrophages did not involve the late endocytic pathway.

**Table 2:** Lack of colocalization of APMV particles with Lamp-1 and Lysotracker red DND99.

Time	Percentage of colocalization with	
	Lamp-1	Tracker red DND99
15 min	0±0	0±0
30 min	0±0	0±0
1 h	0±0	0±0
2 h	0±0	2±1
4 h	0.5±0.025	1±0.5
6 h	2±0.50	1.35±0.75
8 h	1±0.75	0.25±0.003

RAW 264.7 macrophages were incubated with APMV particles (50 PFU/cell) for different periods. The number of APMV particles that colocalized with Lamp-1 and Lysotracker red DND99 was scored. The results are expressed as the percentage of AMPV particles that colocalized with Lamp-1 or Lysotracker red DND99 and are the mean±SD of 3 experiments.

### Ultrastructural analysis of APMV internalization by macrophages

**Figure 7:**
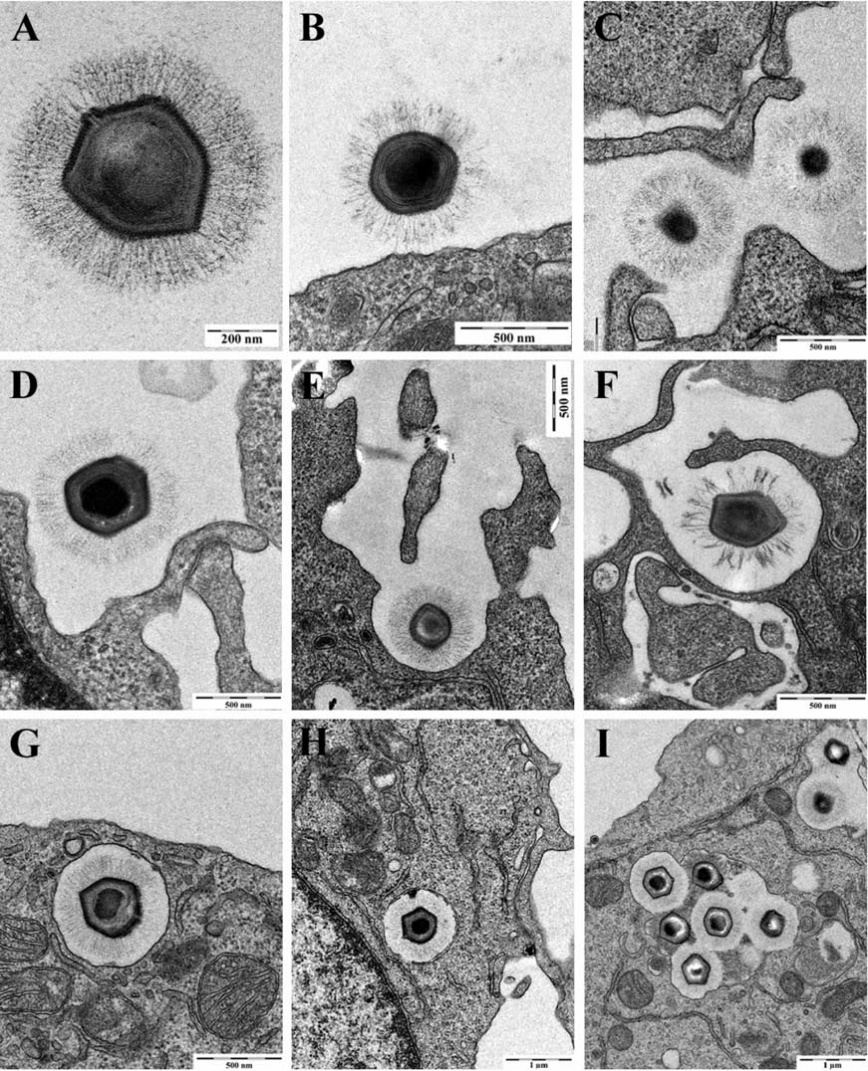
Electron microscopic analysis of APMV internalization. APMV (500 PFU per macrophage) was incubated with RAW 264.7 macrophages for different periods. (A) Isolated APMV. (B) APMV bound to the cell body. (C) APMV attached to cellular extensions. (D and E) Cup-like indentation formed at the cell surface and large cellular extensions starting to embrace APMV. (F) Engulfed APMV. (G) Large smooth-surfaced endocytic vesicle containing APMV. (H) APMV-containing vesicle deeper in the cytoplasm. (I) APMV-containing vesicles that occasionally fused with each other.

Since endocytic pathways such as clathrin-, caveolin- and degradative endosome-mediated endocytosis were not involved in APMV entry, we asked whether APMV used macropinocytosis or phagocytosis to invade macrophages. The uptake of APMV by RAW 264.7 macrophages was studied by electron microscopy. Since APMV particles are large, this study was performed without immunolabeling (**Figure 7A**). Viruses were bound directly at the cell body (**Figure 7B**) or attached to elongated cellular extensions (**Figure 7C**). Then, cup-like indentations were formed at the cell surface (**Figure 7D**), large cell extensions started to embrace viral particles (**Figure 7E**) and, ultimately, completely surrounded these particles (**Figure 7F**). Subsequently, a large smooth-surfaced endocytic vesicle was detected under the plasma membrane (**Figure 7G**). The internalization process of APMV was rapid since viral particles were frequently found within 5 min. Later, endocytic vesicles were found deeper in the cytoplasm (**Figure 7H**) and, occasionally, fused with each other (**Figure 7I**). The morphology of the internalized vesicles did not obviously change up to 3 hours post-infection (p.i.) (data not shown). As the formation of protrusions is usually associated with macropinocytosis and/or phagocytosis, our results suggest that APMV infects macrophages through macropinocytosis or phagocytosis.

### Role of the actin cytoskeleton in APMV entry

**Figure 8:**
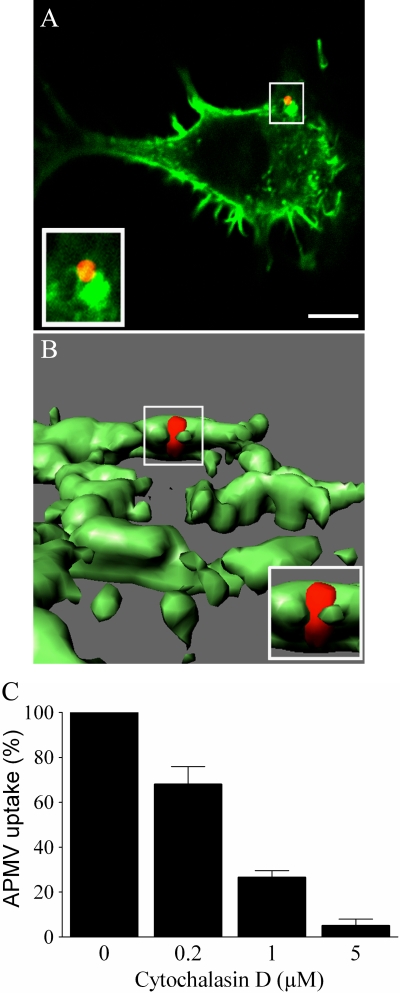
Role of F-actin in APMV internalization. (A) RAW 264.7 macrophages were incubated with APMV particles (50 PFU/cell) for 5 min. F-actin distribution was studied using bodipy phallacidin (green) and APMV localization was detected by immunofluorescence (red). (B) 3D reconstruction of the precedent image. The images are representative of 3 independent experiments. (C) Macrophages were treated with different concentrations of cytochalasin D for 30 min and incubated with APMV particles for 6 hours. Viral particles were visualized by immunofluorescence. The results, expressed as the percentage of APMV uptake relative to the control, are the mean±SD of 3 experiments. Scale bars represent 3 µm.

Since actin polymerization is required for the phagocytic and macropinocytosis cup formation [6],[25], we examined the role of the actin cytoskeleton on APMV uptake by RAW 264.7 macrophages. First, the interaction of APMV particles with filamentous actin (F-actin) was investigated using Alexa Fluor 488-phalloidin and confocal microscopy. F-actin accumulated at the site of APMV entry (**Figure 8A**). About 90% of APMV particles were associated with F-actin accumulation after a 5-min incubation with macrophages. A 3D reconstruction showed that F-actin surrounded viral particles (**Figure 8B** **and Video S1**). Second, RAW 264.7 macrophages were pretreated with cytochalasin D, an inhibitor of actin polymerization, for 30 min and then infected with APMV particles for 6 hours in the presence of cytochalasin D. Viral uptake was determined by immunofluorescence. Cytochalasin D inhibited APMV entry in a dose-dependent manner: in the presence of 0.2 and 1 µM of cytochalasin D, infection was decreased by 32±8% and 73±3%, respectively, and cytochalasin D at 5 µM completely inhibited APMV uptake (**Figure 8C**). The inhibition of APMV entry was not due to a toxic effect of cytochalasin D on macrophages, since cytochalasin D did not affect macrophage viability (**Table 1**). These results show that APMV uptake by macrophages is related to the reorganization of F-actin cytoskeleton.

### Role of phosphatidylinositol 3-kinase (PI3K) pathway in APMV uptake

**Figure 9:**
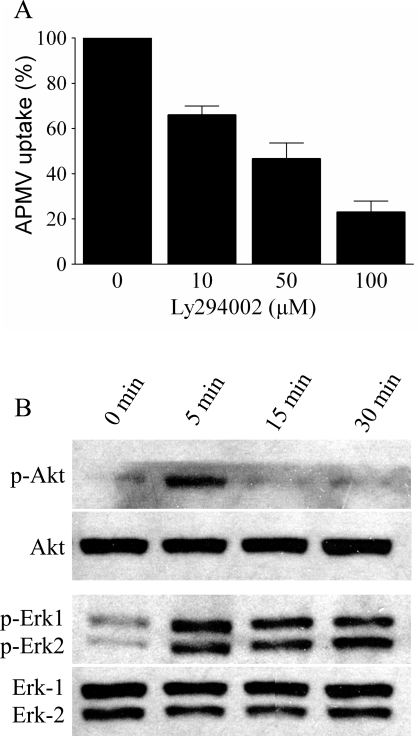
Role of the PI3K pathway in APMV internalization. (A) RAW 264.7 Macrophages were pretreated with different concentrations of LY294002 for 30 min and incubated with APMV particles for 6 hours in the presence of LY294002. Viral particles were visualized by immunofluorescence. The results, expressed as the percentage of APMV uptake relative to the control, are the mean±SD of 3 experiments. (B) Immunoblotting of macrophages stimulated with APMV particles (50 PFU/cell) for different periods was performed with antibodies specific for phosphorylated Akt and ERK. Membranes were stripped and reprobed with anti-Akt and -ERK antibodies, respectively. Each blot is representative of three distinct experiments.

Since PI3Ks are known to be involved in macropinocytosis and phagocytosis, we examined the potential role of PI3Ks in APMV entry. RAW 264.7 macrophages were pretreated with LY294002, a specific inhibitor of PI3Ks, for 30 min, then infected with APMV for 6 hours in the presence of LY294002, and viral entry was determined by immunofluorescence. LY294002 inhibited the internalization of APMV in a dose-dependent manner (**Figure 9A**). This inhibition was not due to a toxic effect of LY294002 since viability of RAW 264.7 cells was not affected by LY294002 (**Table 1**). We also studied the phosphorylation of kinases such as Akt and ERK. Akt was phosphorylated after 5 min of macrophage exposure to APMV. The phosphorylation of Akt was transient since it was undetectable thereafter (**Figure 9B**). ERK-1 and ERK-2 were also phosphorylated after 5 min of stimulation; however, their activation was sustained for at least 1 hour (**Figure 9B**). Taken together, these results show that the PI3K pathway was involved in APMV entry into macrophages.

### Macropinocytosis is not involved in APMV internalization

**Figure 10:**
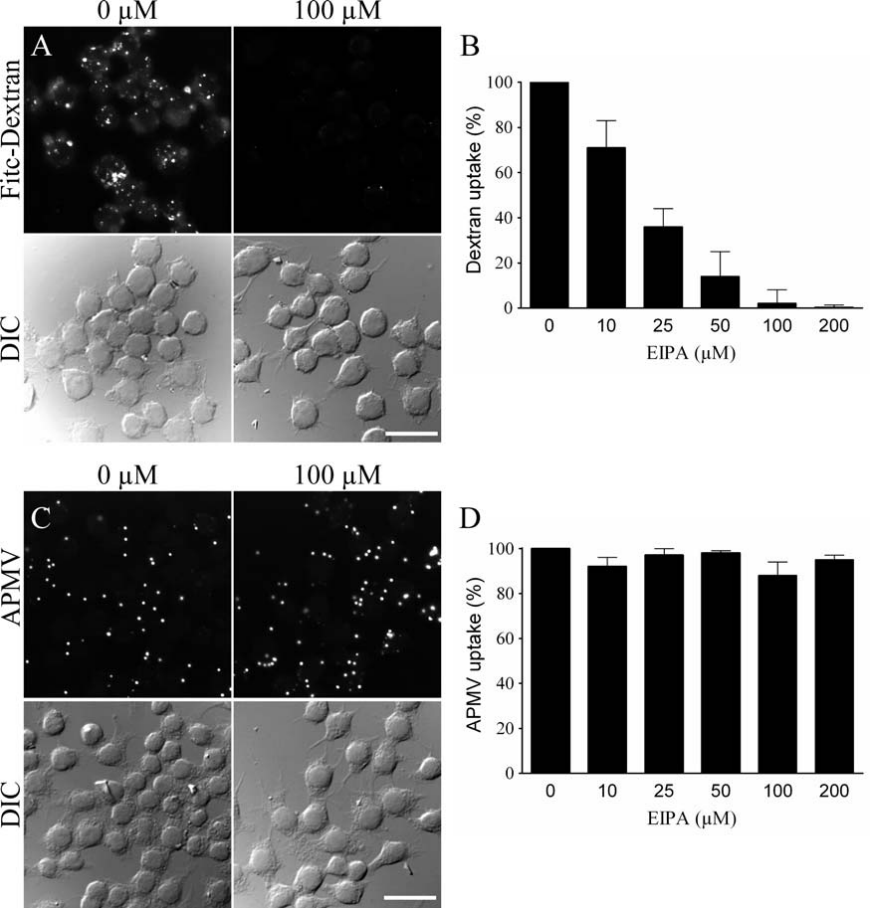
Macropinocytosis is not involved in APMV internalization. (A) RAW 264.7 macrophages were pretreated with 100 µM EIPA for 30 min and incubated with 3 mg/ml FITC-dextran for 30 min. The intracellular distribution of fluorescent dextran was studied in control macrophages (left panels) and in EIPA-pretreated macrophages (right panels). (B) Macrophages were pretreated with different concentrations of EIPA and incubated with FITC-dextran. The results, expressed as the percentage of dextran uptake relative to the control, are the mean±SD of 5 experiments. (C) Macrophages were pretreated with 100 µM EIPA for 30 min before a 6-hour infection with APMV (50 PFU/cell). APMV particles were visualized by indirect immunofluorescence. Their intracellular distribution was studied in control macrophages (left panels) and in EIPA-pretreated macrophages (right panels). (D) Macrophages were pretreated with different concentrations of EIPA and incubated with APMV particles for 6 hours in presence of EIPA. Viral particles were visualized by immunofluorescence. The results, expressed as the percentage of APMV uptake relative to the control, are the mean±SD of 5 experiments. Scale bars represent 50 µm.

**Figure 11:**
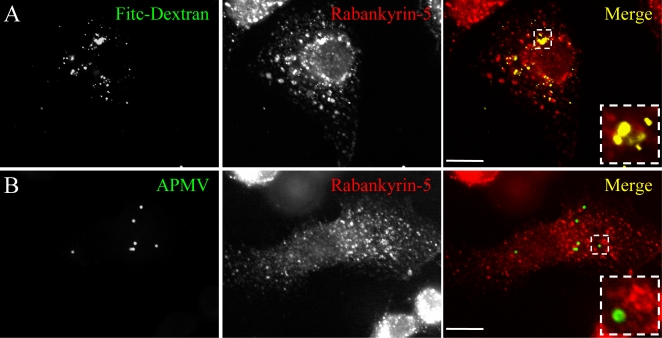
Rabankyrin-5 does not colocalize with APMV. (A) RAW 264.7 macrophages were incubated with 3 mg/ml FITC-dextran for 30 min (top panel). Rabankyrin-5 was revealed using specific antibodies (middle panel). The colocalization of FITC-dextran and rabankyrin-5 was demonstrated by merging fluorescent images (bottom panel). (B) Macrophages were infected with APMV (50 PFU/cell) for 30 min (top panel). Rabankyrin-5 was revealed using specific antibodies (middle panel). The lack of colocalization of APMV particles with rabankyrin-5 was demonstrated by merging fluorescent images (bottom panel). Scale bars represent 5 µm.

To investigate the role of macropinocytosis in the entry of APMV into macrophages, we studied the effect of an inhibitor of macropinocytosis on APMV entry and the colocalization of APMV with rabankyrin-5, a marker of macropinocytosis. 5-ethyl-N-isopropyl amiloride (EIPA) is a specific inhibitor of macropinocytosis that blocks Na^+^/H^+^ exchange [14],[15]. First, EIPA activity was tested through the inhibition of FITC-dextran uptake [14]. EIPA at 10 µM partially inhibited FITC-dextran uptake with complete inhibition at 100 µM (**Figure 10A** **and** **Figure 10B**). Second, RAW 264.7 macrophages were pretreated with different doses of EIPA for 30 min, infected with APMV for 6 hours in the presence of EIPA, and APMV uptake was determined by immunofluorescence. APMV uptake was not affected by EIPA, regardless of its concentration (**Figure 10C** **and** **Figure 10D**). In these experimental conditions, EIPA between 10 and 100 µM did not affect the viability of macrophages (**Table 1**). Third, we studied the colocalization of APMV with rabankyrin-5, a marker of macropinocytosis, using FITC-dextran (MW, 500.000 Da) as a positive control [16]. After 5 min, FITC-dextran fully colocalized with rabankyrin-5 (**Figure 11A**). In contrast, APMV particles did not colocalize with rabankyrin-5 (**Figure 11B**). The absence of colocalization of APMV with rabankyrin-5 was not due to delayed acquisition of rabankyrin-5, since APMV and rabankyrin-5 did not colocalize over a period of 5 min to 6 hours (data not shown). Taken together, these results showed that APMV particles did not enter macrophages through macropinocytosis, suggesting a role for phagocytosis in APMV entry.

### Role of phagocytosis in APMV internalization

**Figure 12:**
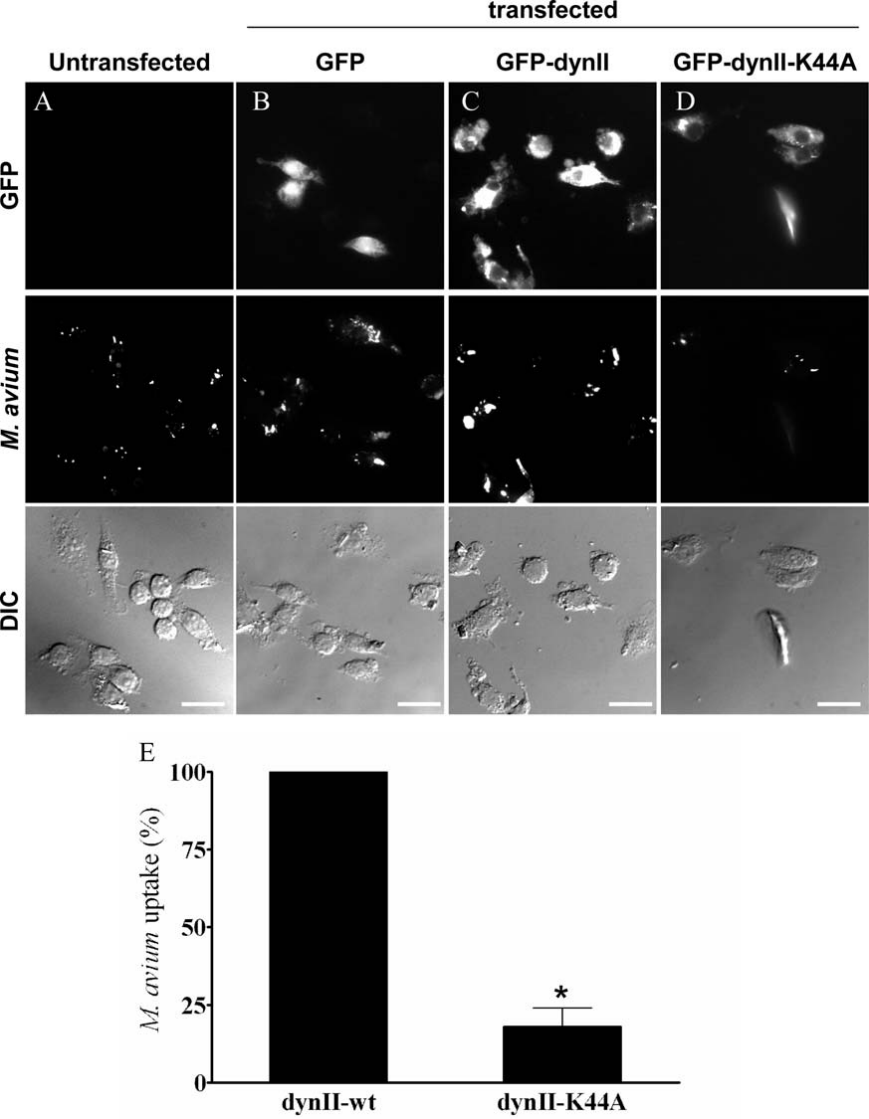
Role of dynamin-II in *M. avium* internalization. RAW 264.7 macrophages (A), macrophages transiently transfected with GFP (B), GFP-tagged active (C) or dominant-negative (D) dynamin-II were infected with Texas red-labelled *M. avium* (10 bacteria/cell) for 6 hours. Fluorescent organisms were visualized by epifluorescence (middle panels). Bacteria were not associated with macrophages transfected with the dominant-negative mutant of dynamin-II. (E) The number of internalized bacteria was scored. The results, expressed as the percentage of bacterial uptake relative to the control, are the mean±SD of 4 experiments. Scale bars represent 25 µm. *p<0.05.

**Figure 13:**
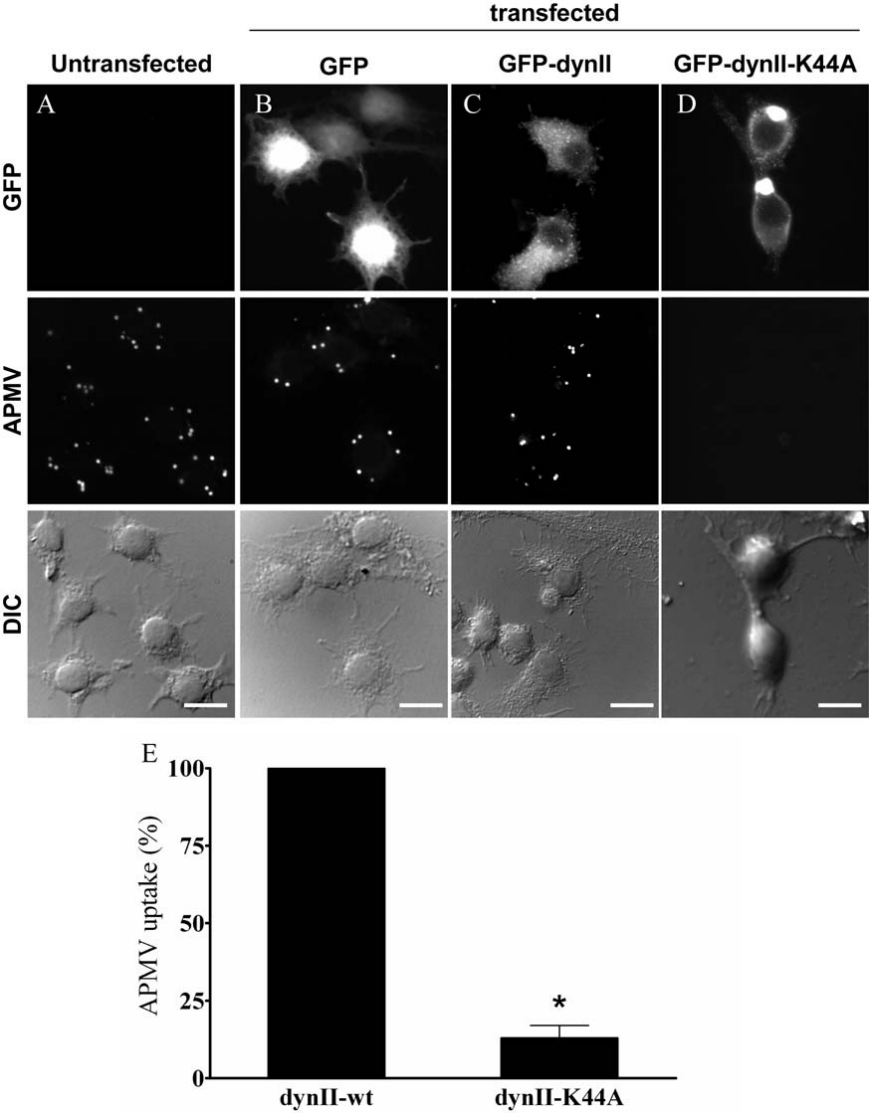
Role of dynamin-II in APMV internalization. RAW 264.7 macrophages (A), macrophages transiently transfected with GFP (B), GFP-tagged active (C) or dominant-negative (D) dynamin-II were infected with APMV (50 PFU/cell) for 6 hours. Viral particles were visualized by immunofluorescence (middle panels). Viral particles were not associated with macrophages transfected with the dominant-negative mutant of dynamin-II. (E) The number of APMV particles internalized was scored. The results, expressed as the percentage of APMV uptake relative to the control, are the mean±SD of 3 experiments. Scale bars represent 50 µm. *p<0.05.

Since phagocytosis, but not macropinocytosis, requires dynamin-II, we investigated the role of dynamin-II in APMV uptake by immunofluorescence. RAW 264.7 macrophages were transfected with GFP-tagged dynamin-II (dynII-wt), a dominant-negative variant of dynamin-II (dynII-K44A), and GFP as a control. First, we controlled that dynamin-II was not involved in other endocytic pathways than phagocytosis [26]. Indeed, we investigated the involvement of dynamin-II in clathrin-mediated endocytosis through the transferrin uptake inhibition (**Figure S2**). The expression of GFP had no effect on the ability of macrophages to internalize transferrin (**Figure S2A and Figure S2B**). Macrophages transfected with dynII-wt (**Figure S2C**) or dynII-K44A (**Figure S2D**) also internalized transferrin (**Figure S2E**), demonstrating that dynamin-II is not required for clathrin-mediated endocytosis. We also investigated the role of dynamin-II in macropinocytosis through the inhibition of dextran uptake (**Figure S3**). The expression of GFP had no effect on the ability of macrophages to internalize fluorescent dextran (**Figure S3A and Figure S3B**). Macrophages transfected with functional dynamin-II (**Figure S3C**) or dynII-K44A (**Figure S3D**) internalized dextran in a similar way (**Figure S3E**) demonstrating that dynamin-II is not required for macropinocytosis. Second, the role of dynamin-II in the phagocytosis process was checked using *Mycobacterium avium*, a bacterium known to enter macrophages through phagocytosis [27]. The expression of GFP had no effect on the ability of macrophages to internalize *M. avium* (**Figure 12A** **and** **Figure 12B**): 89±7% of organisms were internalized by GFP-transfected macrophages compared to untransfected cells. Macrophages transfected with dynII-wt also internalized *M. avium* (**Figure 12C**). In contrast, *M. avium* internalization was significantly (p<0.05) decreased in macrophages transfected with dynII-K44A (**Figure 12D**) as compared to macrophages expressing a functional dynamin-II, since only 18±6% of bacteria were internalized by macrophages transfected with dynII-K44A (**Figure 12E**). These results clearly show that dynamin-II was required for phagocytosis. Third, the role of dynamin-II in APMV entry was investigated. The expression of GFP had no effect on the ability of macrophages to internalize APMV (**Figure 13A** **and** **Figure 13B**): 90±5% of APMV particles were internalized by GFP-expressing macrophages as compared to untransfected cells. Macrophages transfected with functional dynamin-II also internalized APMV (**Figure 13C**). In contrast, macrophages transfected with the dominant-negative variant of dynamin-II did not internalize APMV (**Figure 13D**). In these cells, only 13±4% of APMV particles were associated with macrophages as compared to macrophages that expressed a functional dynamin-II (p<0.05) (**Figure 13E**). These results clearly show that the entry of APMV within macrophages occurs through phagocytosis.

## Discussion

Different internalization pathways are used by viruses (**Figure 1**), including clathrin- or caveolae-mediated endocytosis, macropinocytosis, phagocytosis-like and other endocytic pathways that are poorly characterized such as degradative endosome-mediated endocytosis or phagocytic-like process [9],[28]. To our knowledge, no virus has yet been described to enter professional phagocytes by phagocytosis, a mechanism used by phagocytic cells to ingest particles of more than 0.5 µm or by bacteria and parasites to infect eukaryotic cells. APMV is able to multiply rapidly in *Acanthamoeba polyphaga*, a free living amoebae that possesses major phagocytic activity [2]. It was previously found that microorganisms that replicate in amoeba, such as bacteria or yeasts, should be able to survive to the microbicidal activity of macrophages [29]. The role of professional phagocytes, namely monocytes, macrophages and neutrophils, is to eliminate invading microorganisms through phagocytosis, an active and highly regulated mechanism that involves specific cell surface receptors and signaling cascades mediated by the Rho family of GTPases [6],[19]. It has been recently shown that a phagocytosis-like process is used by herpes simplex virus, a relatively large virus, to infect fibroblastic cells, which are non-professional phagocytes [21]. Several lines of evidence showed that APMV entered macrophages via a phagocytosis process. First, APMV particles entered different professional phagocytes but were unable to infect non-professional phagocytes, although we cannot exclude the hypothesis that a macrophage specific receptor is needed for APMV entry. Moreover, APMV uptake by phagocytes led to a productive cycle of functional viruses. Second, the engulfment of APMV required F-actin. Indeed, cytochalasin D, which inhibits actin polymerization, blocked APMV entry in macrophages in a dose-dependent manner. F-actin labeling with a specific probe and ultrastructure analysis showed cell membrane protrusions at the entry site of APMV and 3D-image reconstruction demonstrated a progressive engulfment of viral particles. Similar indentations and F-actin involvement have been described to be associated with macropinocytosis or phagocytosis events [16],[19], suggesting that APMV internalization could occur through macropinocytosis or phagocytosis. Third, we clearly showed that APMV did not enter macrophages through macropinocytosis since EIPA, a specific inhibitor of macropinocytosis, did not affect APMV entry into macrophages. Furthermore, rabankyrin-5, a rab5 effector that localizes in large vacuolar structures corresponding to macropinosomes [16], did not colocalize with APMV. Fourth, activation of the PI3K pathway mediates multiple cellular functions, including phagocytosis [19]. We showed that APMV activated PI3Ks, since a specific inhibitor of PI3Ks, LY294002, blocked APMV entry into macrophages. In addition, APMV activated downstream effectors, such as Akt and ERK.

The classical endocytic pathway might be involved in or contribute to AMPV entry into macrophages, since caveolae- and clathrin-dependent endocytic routes, known to be used by viruses to infect cells, are also involved in the phagocytic process [11],[12]. First, we found that APMV did not colocalize with caveolin-1, suggesting that a caveolae-dependent pathway was not involved in APMV entry. Second, we found that APMV colocalized with clathrin, but the prevention of clathrin-coated pits by chlorpromazine and the over-expression of a dominant negative mutant of Eps15 did not affect APMV uptake by macrophages. It has also been demonstrated that *Chlamydia* colocalize with clathrin, but the knock-down of clathrin does not affect the bacterial entry [11],[12]. Clearly, these results exclude a role for the clathrin-dependent endocytic routes in APMV uptake by macrophages. Degradative endosome-mediated endocytosis [9] may be also involved in APMV uptake by macrophages. However, APMV did not colocalize with Lamp-1 and lysotracker red DND99, suggesting that the degradative endosome-mediated endocytosis was not involved in APMV internalization. Finally, we showed direct evidence that APMV entered macrophages through phagocytosis. We focused on dynamin-II because it is essential for phagocytosis, but not for macropinocytosis [6],[20],[26] or clathrin-mediated endocytosis [26]. Dynamin-II is essential for the formation of macrophage phagosomes and functions at the stage of membrane extension around the particle [20]. We showed that macrophages transfected with a dominant-negative mutant of dynamin-II, which cannot bind or hydrolyze GTP, exhibited decreased APMV uptake, whereas the active form of dynamin-II enabled APMV entry. However, we cannot rule out a contribution of a lipid raft, caveolin-independent mechanism in APMV entry [30].

**Figure 14:**
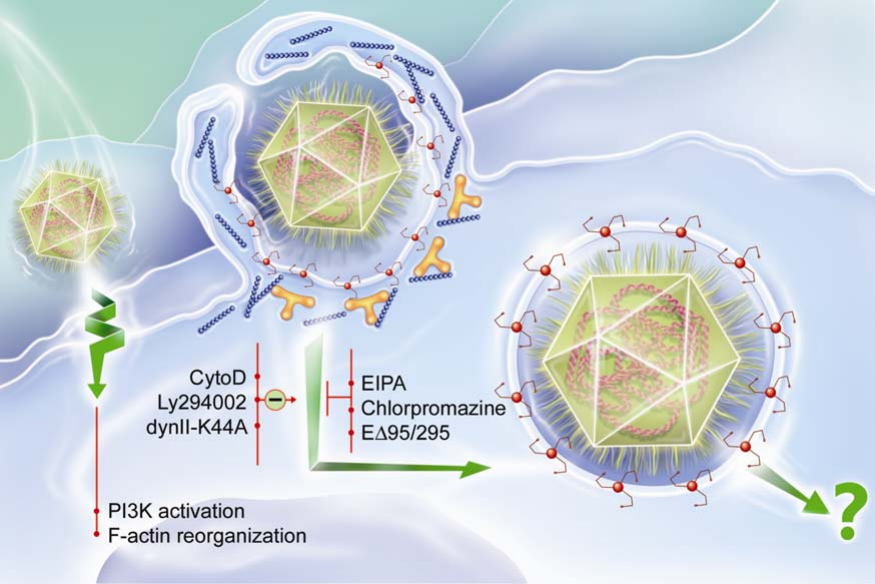
APMV enters macrophages through a phagocytic process. After binding with macrophages, AMPV induces PI3K activation and F-actin polymerization. AMPV particules are engulfed by a mechanism invoving F-actin (blue), clathrin (red) and dynamin-II (yellow). APMV internalization is inhibited by cytochalasin D (CytoD), Ly294002 and the dominant-negative form of the dynamin-II (dynII-K44A), but is not affected by EIPA, chlorpromazine and dominant-negative form of Eps15 (EΔ95/295). The mechanism of APMV replication remains unknown.

Our results demonstrate that the giant virus APMV penetrated macrophages through a phagocytic process normally used by bacteria or parasites (**Figure 14**). Our finding adds one supplementary pathway to already known strategies of virus to enter cells (**Figure 1**). APMV uptake by macrophages led to a productive viral cycle, suggesting that APMV used a strategy not previously reported to survive and replicate within host cells. One of the most intriguing observations in this study is that the process of APMV internalization by macrophages closely resembles APMV internalization by amoebae at the cellular level [5]. As a Trojan horse, free-living amoebae might play a role as a reservoir for intracellular bacteria, in the transmission of pathogens, the selection of virulence traits and in the adaptation of bacteria to macrophages. In support of this, several amoebae-resistant microorganisms, such as *Legionella pneumophila*, *Coxiella burnetii*, *Parachlamydiaceae* and *Cryptococcus neoformans* [29],[31], which are fastidious intracellular bacteria and emerging pathogens, have been shown to be pathogens for macrophages. However, this is the first demonstration that a virus pathogen for amoeba might be also a pathogen for macrophages. In our study, in addition to demonstrating that APMV infects macrophages through phagocytosis, we highlight the fact that APMV is a pathogen for macrophages as it is for amoebae [2], suggesting that phagocytic cells are a human target of APMV. Comparative studies of traits permissive for amoebae and macrophage survival may reveal additional insights into the pathogenesis of APMV infection. We can hypothesize that APMV replicates within alveolar macrophages, leading to human and murine pneumonia. This may help to develop new therapeutic approaches for pneumonia resistant to current treatments.

## Materials and Methods

### APMV preparation

APMV particles were isolated and purified from infected amoebae as previously described [1]. Briefly, APMV-rich-supernatants of infected amoebae were collected and viruses were purified on gastrographin. Purified viruses were then suspended in PBS before being stored at −80°C. The viral titre was determined as follows. Serial dilutions of APMV particles were incubated with 10^5^ amoebae per well in 24-well plates containing Page's modified Neff's amoeba saline [32]. The plates were incubated at 32°C and cells were examined daily to determine amoebal lysis [3]. Amoebal cocultures were fixed with 10% formaldehyde and subsequently stained with 1% crystal violet. The results are expressed as plaque-forming units (PFU)/ml.

### Cell culture

Human monocytes were isolated from peripheral blood mononuclear cells and differentiated into macrophages, as previously described [33]. Mouse BMDM were obtained from a 7-day culture of bone marrow cells in RPMI 1640 containing 10% fetal calf serum (FCS) and 15% of a supernatant of L929 cells rich in Macrophage Colony-Stimulating Factor [34]. Human monocyte THP-1 cells (ATCC N° TIB-202) were also grown in RPMI 1640 containing 10% FCS. Mouse RAW 264.7 macrophages (American Type Culture Collection, ATCC N° TIB-71) and J774A.1 macrophages (ATCC N° TIB-67), and human epithelial A431 cells (ATCC N° CRL-1555) were grown in Dulbecco's Modified Eagle Medium (DMEM) high glucose containing 10% FCS. Mouse neuronal Neuro 2A cells (ATCC N° CCL-131), mouse fibroblast L929 cells (ATCC N° CCL-1), human lung fibroblast HEL299 cells (ATCC N° CCL-137) and human epithelial HeLa cells (ECACC 93021013) were grown in minimum essential medium (MEM) containing 10% FCS. All media were supplemented with 2 mM L-glutamine, 100 UI/ml penicillin and 100 µg/ml streptomycin and were purchased from Invitrogen Life Technologies (Eragny, France). Cell viability assays were performed using the EZ4U kit (Biomedica) according to the manufacturer's recommendations. This method is based on the capacity of living cells to reduce tetrazolium salts into intensely colored formazan derivatives. Briefly, cells were incubated at 37°C with a solution of tetrazolium salts provided by the manufacturer. After incubation, the conversion tetrazolium salts in red formazan derivatives was measured using a microplate-reader set at 450 nm and 620 nm, as a reference. The cell viability was expressed as percentage compared to control.

### Transient transfection

The GFP-EΔ95/295 plasmid was kindly provided by A. Benmerah (Institute Cochin, Paris, France). Dynamin-II (dyn-wt) and dynamin-II K44A (dyn-K44A) plasmid constructs, both GFP-tagged, were kindly provided by M. Mc Niven (Mayo Clinic and Foundation, Rochester, USA). The GFP empty vectors and GFP-caveolin-1 (GFP-Cav1) constructs were prepared as previously described [35]. RAW 264.7 macrophages were transfected with dyn-wt, dyn-K44A, GFP-EΔ95/295, GFP-Cav1 and GFP plasmid constructs using Nucleofactor (Amaxa Biosystems), according to the manufacturer's recommendations.

### Quantitative real-time PCR (qPCR)

RAW 264.7 macrophages were seeded in 24-well plates (5×10^4^ cells per well) for 16 hours and then infected with APMV (10 to 200 PFU/macrophage) for different periods. After extensive washing of cell preparations to discard unbound viruses, viral uptake was determined by qPCR. In some experiments, macrophages were incubated for an additional period of 30 hours to determine APMV replication cycle. In brief, the macrophage culture supernatant was recovered and pooled with macrophage lysate, and DNA was extracted using the QIAamp DNA MiniKit (Qiagen) according to the manufacturer's instructions. The number of viral DNA copies was calculated using the LightCycler-FastStart DNA Master SYBR Green system (Roche). The selected primers specific for APMV were BCFE (5′-TTATTGGTCCCAATGCTACTC-3′) and BCRE (5′-TAATTACCATACGCAATTCCTG-3′) [1]. In each PCR run, a standard curve was generated using serial dilutions ranging from 10 to 10^8^ viral DNA copies using the LightCycler software (LC-Run version 5.32). Results are expressed as the number of viral DNA copies.

### Immunofluorescence and image analysis

RAW 264.7 macrophages were seeded on 12-mm diameter glass coverslips in 24-well plates (5×10^4^ cells per well) for 16 hours. To determine the role of clathrin, cytoskeleton reorganization, PI3K activation and macropinocytosis in APMV uptake, macrophages were pretreated for 30 min with chlorpromazine, cytochalasin D, LY294002 and EIPA, respectively. All inhibitors were purchased from Sigma-Aldrich (St. Louis, MO). Macrophages were then infected with APMV (50 PFU/cell) for 6 hours, extensively washed to discard unbound viruses and fixed in 3% paraformaldehyde or methanol. Fixed macrophages were permeabilized with 0.1% Triton X-100 and immunofluorescence labelling was performed according to standard procedures [36]. Rabbit and mouse polyclonal antibodies directed against APMV were generated in our laboratory [3]. After fluorescent labelling, macrophages were mounted with Mowiol and examined in fluorescence and differential interference contrast (DIC) modes with an Olympus microscope (Bx511) equipped with a Nikon digital camera (Sight DS5M) using a 60× objective lens. Pictures were processed with Adobe Photoshop V5.5 software. Macrophage infection was scored by examining at least 200 macrophages per experimental condition: 50 microscope fields, with at least 3 macrophages per field containing at least 3 virus, were randomly selected. The percentage of uptake was calculated as the product of the mean number of viral particles per infected macrophage and the percentage of infected macrophages ×100. When chlorpromazine and EIPA were used, the quantification of grey values of thresholded, fluorescent images of at least 20 cells was performed using ImageJ software (NIH, http://rsb.info.nih.gov/ij) [16]. The results are expressed as the percentage of uptake relative to the control.

### Transferrin and dextran uptake

RAW 264.7 macrophages were serum starved, pretreated with chlorpromazine for 30 min, and incubated with 50 µg/ml Alexa 488- or Alexa 555-conjugated transferrin (Molecular Probes) for 15 min at 37°C. To remove external transferrin, macrophages were acid washed (0.1 M glycine, 0.1 M NaCl, pH 3.0), then fixed with 3% paraformaldehyde and mounted with Mowiol. Control cells were processed as described without chlorpromazine pretreatment. Dextran uptake by macrophages was assayed as follows. Macrophages were pretreated with EIPA for 30 min and then incubated with 3 mg/ml FITC- or Alexa 555- conjugated dextran (lysine fixable; MW, 50 kDa, Molecular Probes) for 30 min. After washing, macrophages were fixed with 3% paraformaldehyde and mounted with Mowiol. Control cells were processed as described without EIPA pretreatment. Imaging, and transferrin and dextran uptake were determined as described above.

### Colocalization experiments and F-actin labelling

RAW 264.7 macrophages were seeded on 12-mm diameter glass coverslips in 24-well plates (5×10^4^ cells per well) for 16 hours and then infected with APMV (50 PFU/cell). Rat antibodies specific for Lamp-1 (clone 1D4B) were purchased from DSHB (Iowa, USA), and rabbit antibodies specific for rabankyrin-5 and clathrin were kindly provided by M. Zerial (MPI-CBG, Dresden, Germany) and S. Meresse (CIML, Marseille, France), respectively. Secondary Alexa antibodies were a generous gift from M. Zerial. The distribution of F-actin was studied using a specific probe, Alexa Fluor 488-phalloidin (Molecular Probes), and standard procedures. RAW 264.7 macrophages were incubated with Lysotracker red DND99 (Molecular Probes) at 100 nM for 2 hours and, after washing, they were infected with APMV. After fluorescent labelling, macrophages were mounted with Mowiol and examined as described above. At least 60 macrophages were examined for each experimental condition and results are expressed as the percentage of APMV particles that colocalized with fluorescent markers. Macrophages were selected as following: 50 microscope fields, with at least 3 macrophages per field containing at least 3 viruses, were randomly selected. In some experiments, macrophages were examined by laser scanning microscopy using a confocal microscope (Leica TCS SP2) with a 63X/1.32-0.6 oil CS lens and an electronic Zoom 4X. Optical sections of fluorescent images were collected at 0.25-µm intervals using Leica Confocal Software and processed using Adobe Photoshop V5.5 software or Imaris software for the 3D reconstruction.

### Electron microscopy

RAW 264.7 macrophages (5×10^4^ cells per assay) seeded on glass coverslips were infected with APMV (500 PFU/macrophage). After different periods, they were fixed with 2.5% glutaraldehyde buffered with 50 mM sodium cacodylate, 50 mM KCl and 2.5 mM MgCl_2_ (pH 7.2) for 30 min at room temperature. Samples were rinsed several times with cacodylate buffer and post-fixed with ice-cold 2% osmium tetroxide for 1 hour. After washing in distilled water, the samples were stained with 0.5% uranyl acetate for 18 hours. Dehydration was performed using graded concentrations of ethanol (50–100%) and propylenoxide (100%). Macrophages were embedded in Epon, sectioned and stained with uranyl acetate (2% in methanol) and lead citrate. Samples were examined with a FEI Morgagni 268 (D) transmission electron microscope [37],[38].

### Western blotting

RAW 264.7 macrophages (5×10^6^ cells per assay) were incubated with APMV (50 PFU/macrophage) for different periods at 37°C. Macrophages were washed with cold PBS, scraped and pelleted at 500× *g* for 10 min at 4°C. Western blotting was performed as described elsewhere [39]. Briefly, macrophages were incubated in ice-cold lysis buffer containing 1% Triton X-100, sodium orthovanadate and protease inhibitors (Complete, Roche Diagnostics). Samples were pelleted at 4,000× *g* for 20 min at 4°C. Protein content of supernatants was adjusted and 50 µg proteins were loaded on sodium dodecyl sulfate-12% polyacrylamide gels (SDS-PAGE) under reducing conditions. After electrophoresis, proteins were transferred to nitrocellulose membranes. Unreacted sites were blocked in a solution containing 0.05% Tween 20 and 5% milk for 2 hours. After washing, blots were washed and incubated with a 1∶1,000 dilution of polyclonal antibodies directed against phosphorylated Akt (Ser473) or monoclonal antibodies specific for phosphorylated ERK-1/ERK2 (Thr202/Tyr204) for 60 min. Antibodies were purchased from Cell Signaling. After washing, nitrocellulose blots were incubated with a 1∶1,000 dilution of peroxidase-conjugated F(ab′)_2_ anti-mouse immunoglobulin G (Amersham) for 60 min and visualized using an enhanced chemiluminescence detection kit (ECL, Amersham). Membranes were stripped, reprobed with polyclonal anti-Akt antibodies and anti-ERK antibodies (Cell Signaling), respectively, and incubated with secondary antibodies before ECL visualization.

### *M. avium* preparation and infection of macrophages

*M. avium spp avium* (ATCC N° 25291) was cultured in Middlebrook 7H9 broth (Life Technologies) supplemented with 10% Middlebrook oleic acid, albumin, dextrose, and catalase enrichment (OADC) and 0.2% Tween 80, as previously described [40]. Bacteria were frozen at −80°C in 7H9 medium with 10% OADC. Organisms were labelled with 1 µg/ml Texas red succinimidyl ester (Molecular Probes) according to the manufacturer's protocol [41], and bacterial viability was checked. RAW 264.7 macrophages were seeded on 12-mm diameter glass coverslips in 24-well plates (5×10^4^ cells per well) for 16 hours, then infected with fluorescent organisms (bacterium-to-cell ratio of 10∶1). After 6 hours, cells were extensively washed to discard unbound bacteria and fixed in 3% paraformaldehyde.

### Statistical analysis

The results are given as mean±SD. Student's *t* test analysis was performed using the GraphPad 4 Prism software. Differences were considered significant when *p*<0.05.

## Supporting Information

Figure S1 Inhibition of transferrin uptake by a dominant negative mutant of Eps15. RAW 264.7 macrophages (A), macrophages transiently transfected with GFP (B) and dominant-negative mutant of Eps15 (EΔ95/295) (C) were incubated with 50 µg/ml Alexa 555-conjugated transferrin for 15 min. Transferrin was not internalized by macrophages transfected with EΔ95/295. (D) The transferrin uptake was visualized (middle panels) and quantified. The results, expressed as the percentage of transferrin uptake relative to the control, are the mean±SD of 3 experiments. Scale bars represent 25 µm.
(5.74 MB TIF)

Figure S2 Dynamin-II is not involved in transferrin uptake. RAW 264.7 macrophages (A), macrophages transiently transfected with GFP (B), GFP-tagged active (C) or dominant-negative (D) dynamin-II were incubated with 50 µg/ml of Alexa 555-conjugated transferrin for 15 min. The intracellular distribution of fluorescent transferrin was visualized by epifluorescence (middle panels). Transferrin uptake was not inhibited in macrophages transfected with the dominant-negative mutant of dynamin-II. (E) The results, expressed as the percentage of transferrin uptake relative to the control, are the mean±SD of 3 experiments. Scale bars represent 25 µm.
(7.40 MB TIF)

Figure S3 Dynamin-II is not involved in dextran uptake. RAW 264.7 macrophages (A), macrophages transiently transfected with GFP (B), GFP-tagged active (C) or dominant-negative (D) dynamin-II were incubated with 3 mg/ml of Alexa 555-conjugated dextran for 30 min. The intracellular distribution of fluorescent dextran was visualized by epifluorescence (middle panels). Dextran uptake was not inhibited in macrophages transfected with the dominant-negative mutant of dynamin-II. (E) The results, expressed as the percentage of dextran uptake relative to the control, are the mean±SD of 3 experiments. Scale bars represent 25 µm.
(6.57 MB TIF)

Video S1 3D reconstruction of APMV engulfment by macrophages. RAW 264.7 macrophages were incubated with APMV for 5 min. F-actin distribution was studied with Alexa Fluor 488-phalloidin (green) and APMV localization was determined by immunofluorescence (red). Using a laser-scanning microscope, a series of 22 pictures (Z section) in each fluorescent channel were captured before transfer into Imaris software and 3D reconstruction. The top and bottom of cells were removed with Imaris software to improve the visual quality of the 3D reconstruction.
(2.68 MB MOV)
