# Supplementary figures and images for: Ameobal Pathogen Mimivirus Infects Macrophages through Phagocytosis
Source: PLoS Pathog. 2008 Jun 13;4(6):e1000087. doi: 10.1371/journal.ppat.1000087 (PMC2398789; doi:10.1371/journal.ppat.1000087)

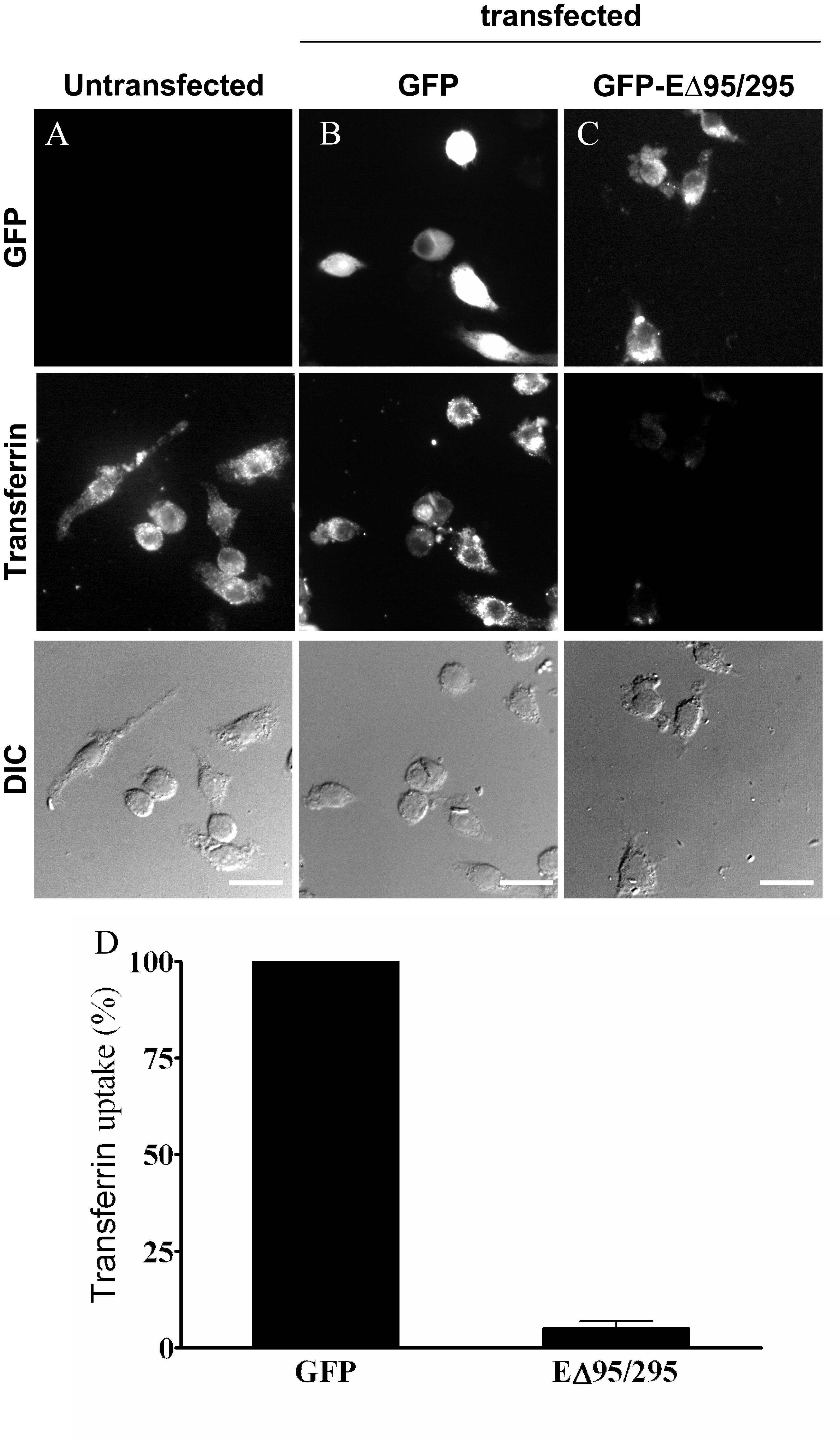

Supplement: Figure S1 — Inhibition of transferrin uptake by a dominant negative mutant of Eps15. RAW 264.7 macrophages (A), macrophages transiently transfected with GFP (B) and dominant-negative mutant of Eps15 (EΔ95/295) (C) were incubated with 50 µg/ml Alexa 555-conjugated transferrin for 15 min. Transferrin was not internalized by macrophages transfected with EΔ95/295. (D) The transferrin uptake was visualized (middle panels) and quantified. The results, expressed as the percentage of transferrin uptake relative to the control, are the mean±SD of 3 experiments. Scale bars represent 25 µm. (5.74 MB TIF) [file ppat.1000087.s001.tif]

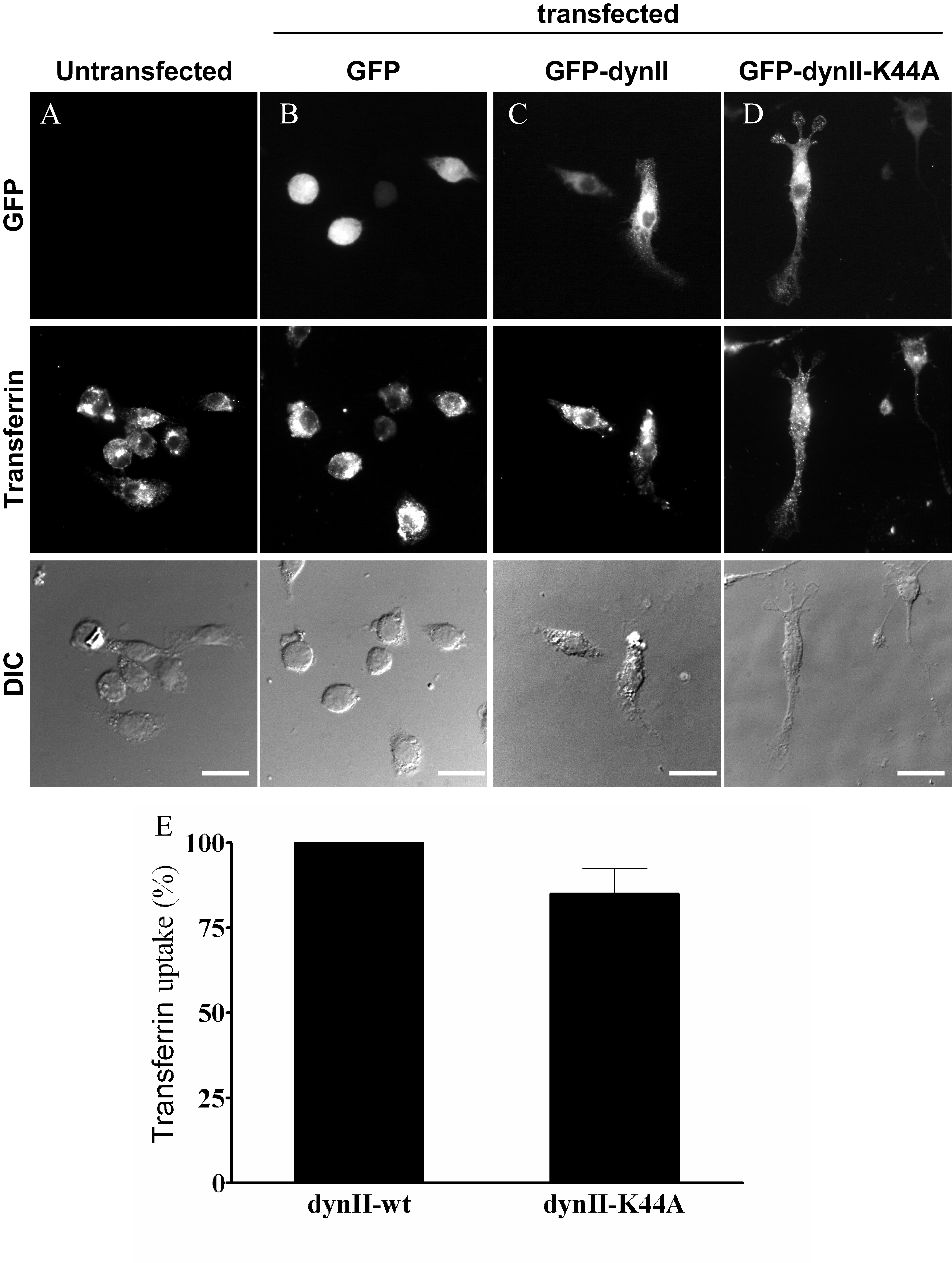

Supplement: Figure S2 — Dynamin-II is not involved in transferrin uptake. RAW 264.7 macrophages (A), macrophages transiently transfected with GFP (B), GFP-tagged active (C) or dominant-negative (D) dynamin-II were incubated with 50 µg/ml of Alexa 555-conjugated transferrin for 15 min. The intracellular distribution of fluorescent transferrin was visualized by epifluorescence (middle panels). Transferrin uptake was not inhibited in macrophages transfected with the dominant-negative mutant of dynamin-II. (E) The results, expressed as the percentage of transferrin uptake relative to the control, are the mean±SD of 3 experiments. Scale bars represent 25 µm. (7.40 MB TIF) [file ppat.1000087.s002.tif]

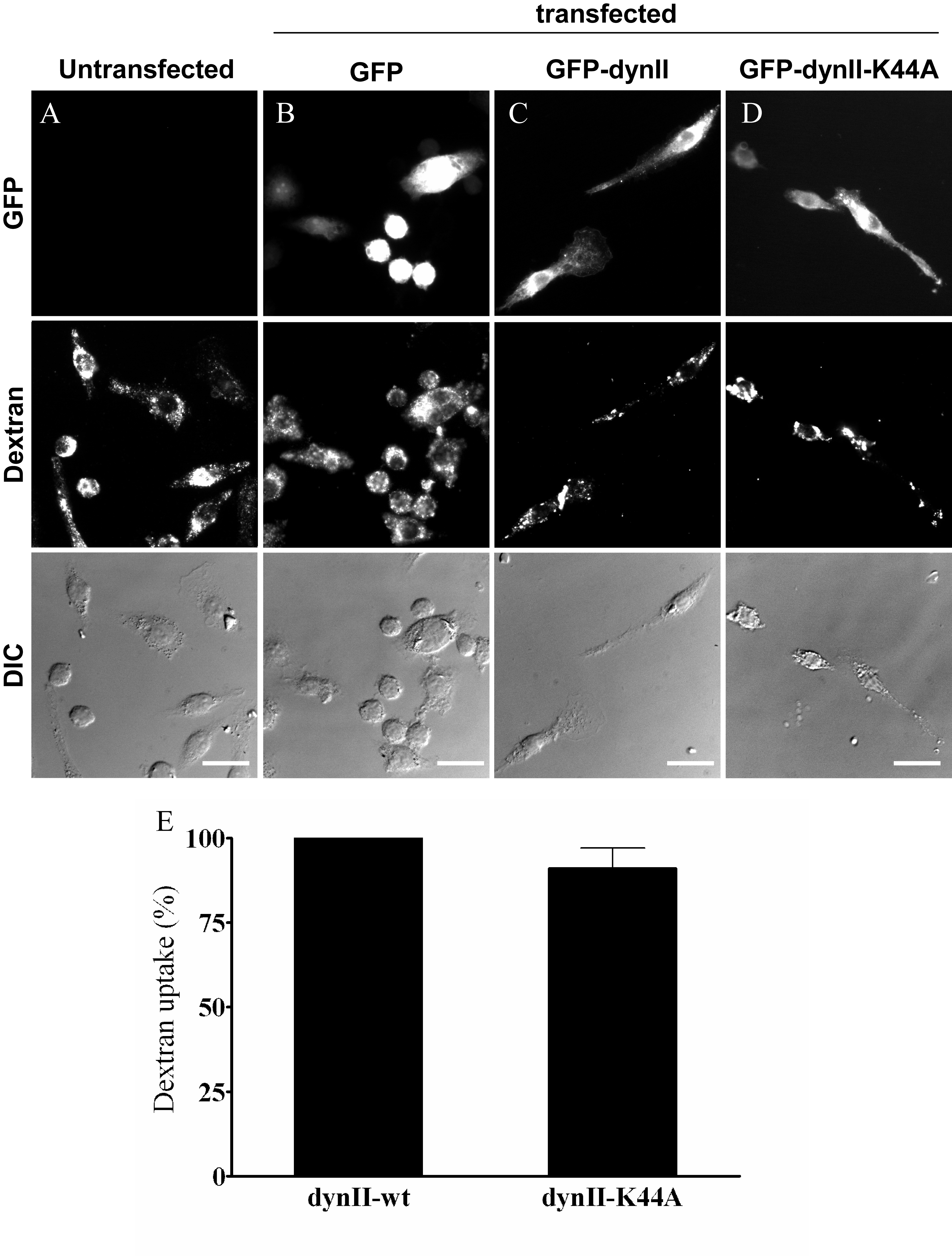

Supplement: Figure S3 — Dynamin-II is not involved in dextran uptake. RAW 264.7 macrophages (A), macrophages transiently transfected with GFP (B), GFP-tagged active (C) or dominant-negative (D) dynamin-II were incubated with 3 mg/ml of Alexa 555-conjugated dextran for 30 min. The intracellular distribution of fluorescent dextran was visualized by epifluorescence (middle panels). Dextran uptake was not inhibited in macrophages transfected with the dominant-negative mutant of dynamin-II. (E) The results, expressed as the percentage of dextran uptake relative to the control, are the mean±SD of 3 experiments. Scale bars represent 25 µm. (6.57 MB TIF) [file ppat.1000087.s003.tif]
